# Supplementary material for: A calcium-based plasticity model for predicting long-term potentiation and depression in the neocortex
Source: Nat Commun. 2022 Jun 1;13:3038. doi: 10.1038/s41467-022-30214-w (PMC9160074; doi:10.1038/s41467-022-30214-w)
Supplement: Supplementary file 1 — Supplementary Figures [file 41467_2022_30214_MOESM1_ESM.pdf]

Supplementary Information

A calcium-based plasticity model for predicting long-term potentiation and depression in the neocortex.

Chindemi *et al.* 2022

A. Supplementary figures

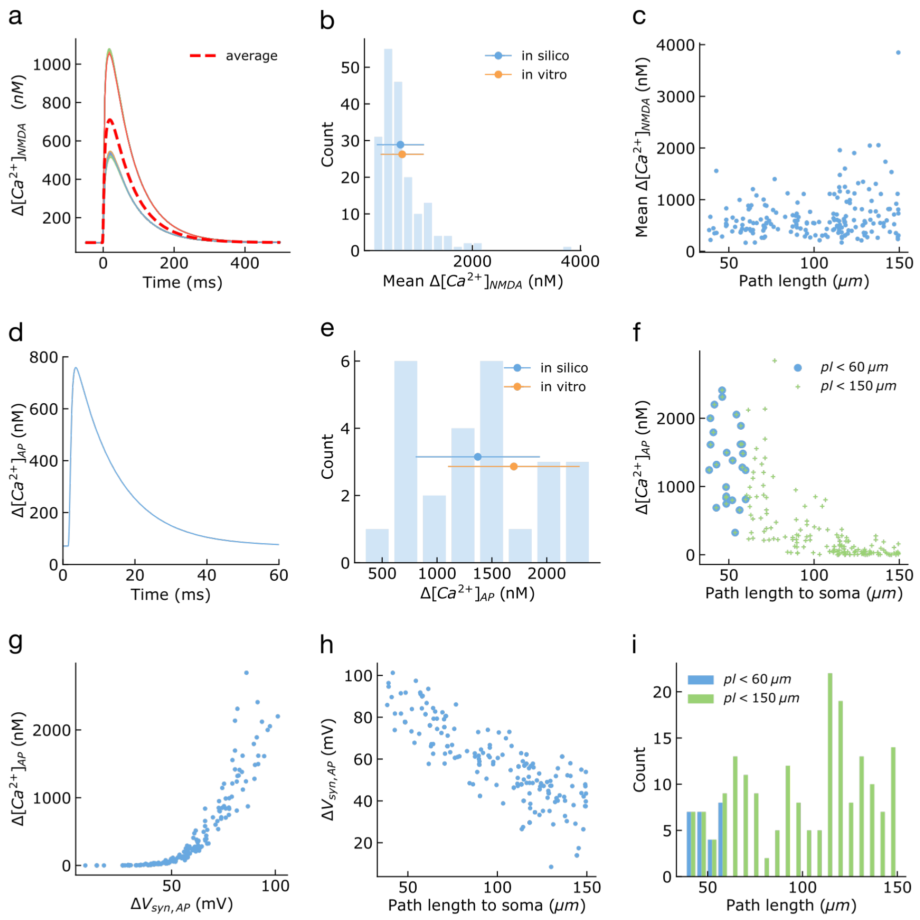

Figure A.1: Validation of calcium dynamics in spines of L5-TTPC to L5-TTPC connections during synaptic events and APs. (**a**) Representative calcium transients due to presynaptic activation at a single synapse overlaid ($n = 21$; synaptic transmission failures excluded; thin solid lines), and their mean (average; thick dashed red line). Small or large peaks indicate release of one or two vesicles, respectively. (**b**) Distribution of mean calcium transient peaks due to presynaptic activation for a population of synapses ($n = 190$). Error bars represent mean and STD of the *in silico* (blue; 0.67 ± 0.44 µm) and *in vitro* (orange; 0.7 ± 0.4 µm) distributions of mean calcium transient peaks. (**c**) Dependence of the mean calcium transient peaks on path length of the stimulated synapse from the soma. (**d**) Representative calcium transient at a single synapse due to one AP. (**e**) Distribution of calcium transient peaks due to one AP for a population of synapses ($n = 26$). Error bars represent mean and STD of the *in silico* (blue; 1.4 ± 0.6 µm) and *in vitro* (orange; 1.7 ± 0.6 µm) distribution of calcium transient peaks. (**f**) Dependence of the calcium transient peaks on path length of the synapse from the stimulated soma. In (e), only synapses closer than 60 µm from soma were considered (see Methods). (**g**) Dependence of the calcium transient peaks on the associated spine voltage transient peaks due to one AP. (**h**) Dependence of the spine voltage transient peaks on path length from the stimulated soma. (**i**) Distribution of path lengths from synapse to soma for the population of spines on dendrites with path length < 60 µm (blue) or < 150 µm (green). All data reported as mean ± STD, *in vitro* STD estimated by propagation of errors. Experimental data (*in vitro*) from Sabatini *et al.*[1].

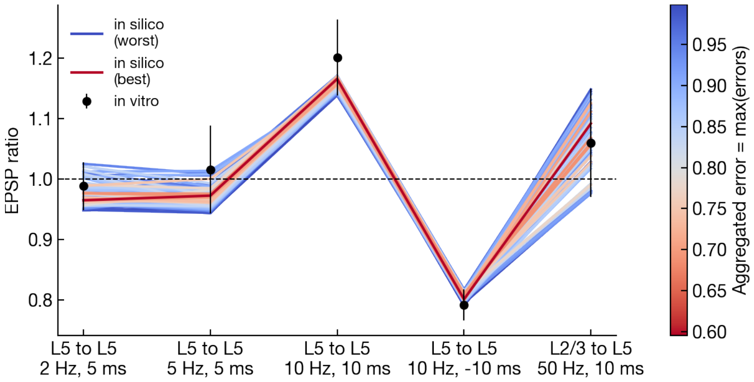

Figure A.2: Performance on the training set of the 82 best candidate solutions. The pool of best candidates is defined as the subset of all evaluated solutions where the mean *in silico* EPSP ratio on every protocol (solid line) is within one SEM of the corresponding *in vitro* target (black error bar, mean ± SEM). Line colors represent the aggregated error of each solution, defined as the maximum of its errors across the target protocols. The best solution is the one minimizing the aggregated error (*in silico* (best); red line). Error bars of candidate solutions were omitted for clarity. Experimental data (*in vitro*) from Markram *et al.*[2], Sjöström & Häusser[3].

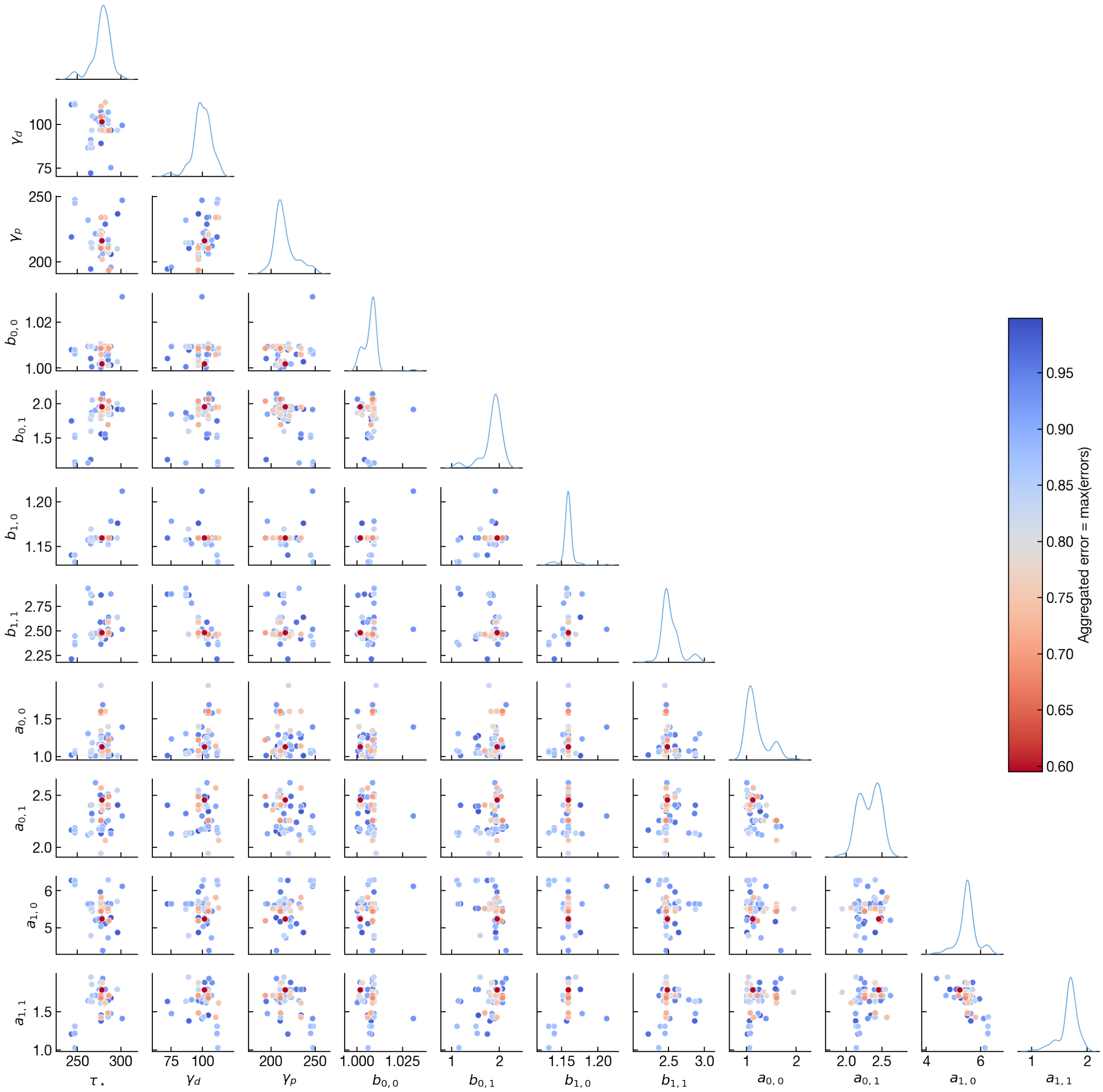

Figure A.3: Plasticity parameter sets and their pairwise relationships for the 82 best candidate solutions. Kernel density estimate (KDE) of each parameter distribution are reported on the main diagonal. Scatter plots show the pairwise relationship between synaptic plasticity parameters. Colors of the markers represent the aggregated error of each solution on the training set, defined as the maximum of its errors across the target protocols. Candidate solutions and color code as in Supplementary Figure A.2

.

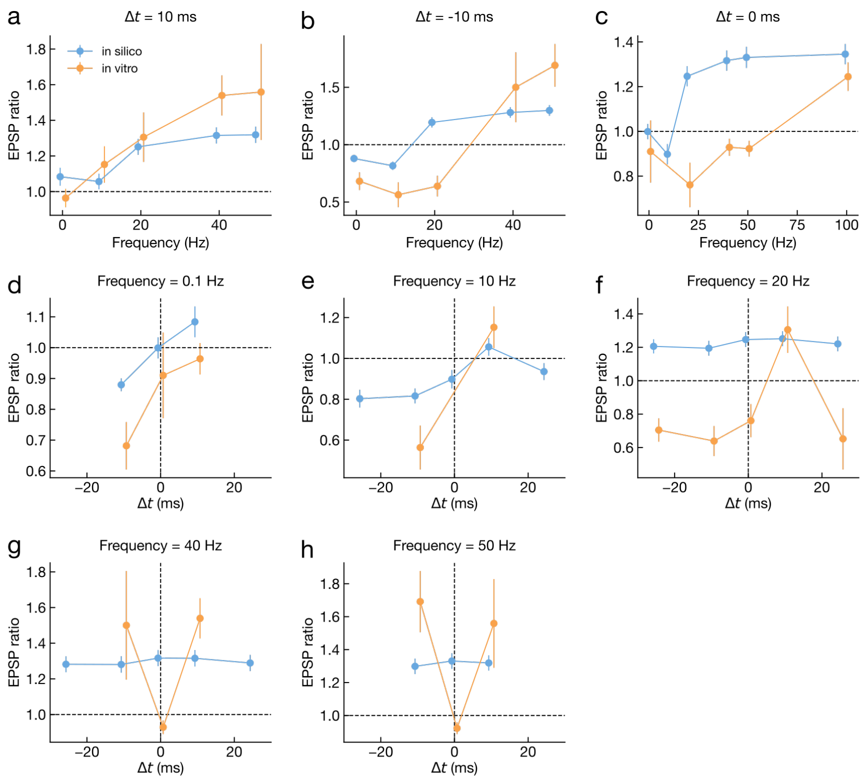

Figure A.4: Comparison of *in vitro* LTP/LTD outcomes in the visual cortex to the *in silico* model for L5-TTPC to L5-TTPC connections. (**a**) Comparison of *in vitro* and *in silico* frequency dependence of synaptic changes at $\Delta t = +10$ ms. Welch's unequal variances two-sided t-test was n.s. for every protocol (*p-value* from low to high stimulation frequency: 0.107, 0.416, 0.741, 0.109, 0.418; $n = 100$). (**b**) Comparison of *in vitro* and *in silico* frequency dependence of synaptic changes at $\Delta t = -10$ ms. Welch's unequal variances two-sided t-test was significant for two out of five protocols (*p-value* from low to high stimulation frequency: 0.043, 0.132, 0.003, 0.514, 0.067; $n = 100$). (**c**) Comparison of *in vitro* and *in silico* frequency dependence of synaptic changes at $\Delta t = 0$ ms. Welch's unequal variances two-sided t-test was significant for three out of five protocols (*p-value* from low to high stimulation frequency: 0.564, 0.005, < 0.001, < 0.001, 0.229; $n = 100$). (**d**) Comparison of *in silico* and *in vitro* STDP at a frequency of 0.1 Hz. Welch's unequal variances two-sided t-test was significant for one out of three protocols (*p-value* from negative to positive stimulation timing: 0.043, 0.564, 0.107; $n = 100$). (**e**) Comparison of *in silico* and *in vitro* STDP at a frequency of 10 Hz. Welch's unequal variances two-sided t-test was n.s for every protocol (*p-value* from negative to positive stimulation timing: 0.132, 0.416; $n = 100$). (**f**) Comparison of *in silico* and *in vitro* STDP at a frequency of 20 Hz. Welch's unequal variances two-sided t-test was significant for four out of five protocols (*p-value* from negative to positive stimulation timing: < 0.001, 0.003, 0.005, 0.741, 0.034; $n = 100$). (**g**) Comparison of *in silico* and *in vitro* STDP at a frequency of 40 Hz. Welch's unequal variances two-sided t-test was significant for one out of three protocols (*p-value* from negative to positive stimulation timing: 0.514, < 0.001, 0.109; $n = 100$). (**h**) Comparison of *in silico* and *in vitro* STDP at a frequency of 50 Hz. Welch's unequal variances two-sided t-test was significant for one out of three protocols (*p-value* from negative to positive stimulation timing: 0.067, < 0.001, 0.418; $n = 100$). At 40 and 50 Hz, the $\Delta t = 0$ ms data points *in vitro* are identical and acquired by pulling experiments together, as described in Sjöström *et al.*[4]. All data reported as mean ± SEM. All experimental data (*in vitro*) from Sjöström *et al.*[4]. For the full distribution of *in silico* experiment outcomes see Supplementary Figure A.20.

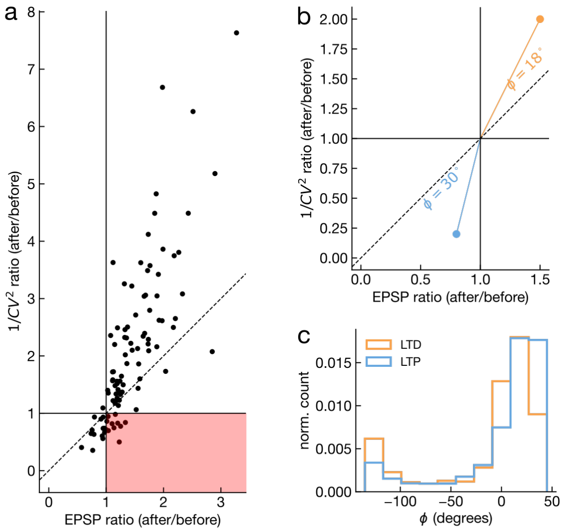

Figure A.5: Analysis of the putative locus of expression of synaptic plasticity in L5-TTPC to L5-TTPC connections. (**a**) CV analysis of a plasticity inducing protocol *in silico* (50 Hz, 5 ms stimulation, same simulation data used in Figure 3 (a-f)). Each symbol represents a L5-TTPC to L5-TTPC connection (n = 100). For an EPSP ratio greater than 1 (solid vertical line), points above the diagonal (dashed line) indicate connections exhibiting a putative presynaptic locus of expression, while points below the diagonal indicate a putative postsynaptic locus of expression. For an EPSP ratio smaller than 1 (solid vertical line), points above the diagonal (dashed line) indicate connections exhibiting a putative postsynaptic locus of expression, while points below the diagonal indicate a putative presynaptic locus of expression. Points in the red shaded area corresponds to connections that underwent putative presynaptic LTD and postsynaptic LTP. Mean and CV of EPSP ratio computed from 60 consecutive EPSPs before and after plasticity induction. (**b**) The angle formed between each point in (**a**) and the diagonal can be used as a summarizing measure of the locus of expression. (**c**) Locus of expression analysis for the whole L5-TTPC to L5-TTPC dataset (same simulation data used in Figure 3 (a-f)). Data analysis procedures adapted from Sjöström *et al.*[5]

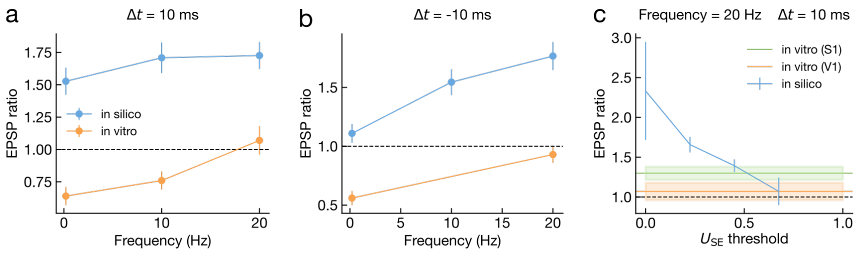

Figure A.6: Comparison of *in vitro* LTP/LTD outcomes in the visual cortex to the *in silico* model for L2/3-PC to L2/3-PC connections. (**a**) Comparison of *in vitro* and *in silico* frequency dependence of synaptic changes at $\Delta t = +10$ ms. Welch's unequal variances two-sided t-test was significant for every protocol (*p-value* < 0.001; $n = 99$, due to an excluded outlier). (**b**) Comparison of *in vitro* and *in silico* frequency dependence of synaptic changes at $\Delta t = -10$ ms. Welch's unequal variances two-sided t-test was significant for every protocol (*p-value* < 0.001; $n = 99$, due to an excluded outlier). (**c**) EPSP ratio dependence on weak connections cut off. Connections whose mean release probability was below the threshold (U_{SE} threshold, x axis) were excluded from the *in silico* EPSP ratio calculations (blue error bars). Corresponding *in vitro* experiments in S1 (green, Egger *et al.*[6]) and V1 (orange, Zilberter *et al.*[7]). All data reported as mean ± SEM. All experimental data (*in vitro*) from Zilberter *et al.*[7] (a-c) and Egger *et al.*[6] (c). For the full distribution of *in silico* experiment outcomes see Supplementary Figure A.21.

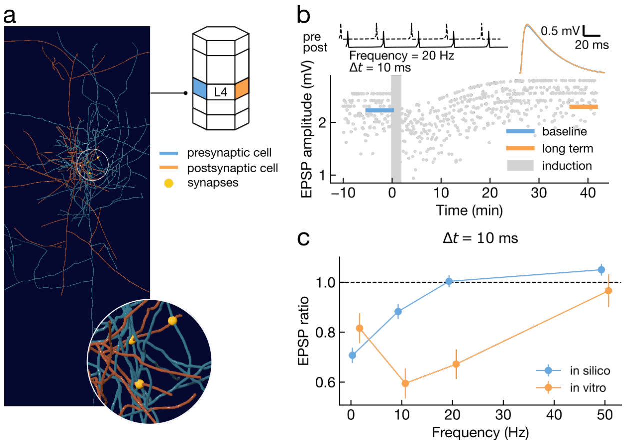

Figure A.7: Testing plasticity model generalization to non-PC connections: L4-SSC to L4-SSC. (**a**) 3-D rendering of a representative pair of connected L4-SSC to L4-SSC in the *in silico* model. Inset shows a magnified view of the synapses mediating the connection (yellow spheres). (**b**) Evolution over time of EPSP amplitude during a typical plasticity induction simulation (bottom). The induction protocol consists of pairs of spikes repeated 100 times at intervals of 5 s (top left; one pair shown). The presynaptic spike follows the postsynaptic one by 15 ms ($\Delta t = -15$ ms). Presynaptic spikes depicted for illustration purposes only (see Methods for details on simulations). Mean EPSP (top right) and peak amplitude (bottom) are shown before (baseline; blue) and after (long term; orange) the induction protocol. In subsequent panels, the change in connection strength is measured as the ratio of the mean EPSP amplitude after the induction protocol to baseline. (**c**) Comparison of *in silico* and *in vitro* frequency dependence of synaptic changes at $\Delta t = 10$ ms. Welch's unequal variances two-sided t-test showed significant differences for two out of four protocols (*p-value* from low to high stimulation frequency: 0.170, 0.005, < 0.001, 0.299; $n = 99$). Experimental data (*in vitro*) from Egger *et al.*[6]. Population data reported as mean ± SEM (n = 99, one connection eliminated because of spiking activity during baseline). For the full distribution of *in silico* experiment outcomes see SI A.22.

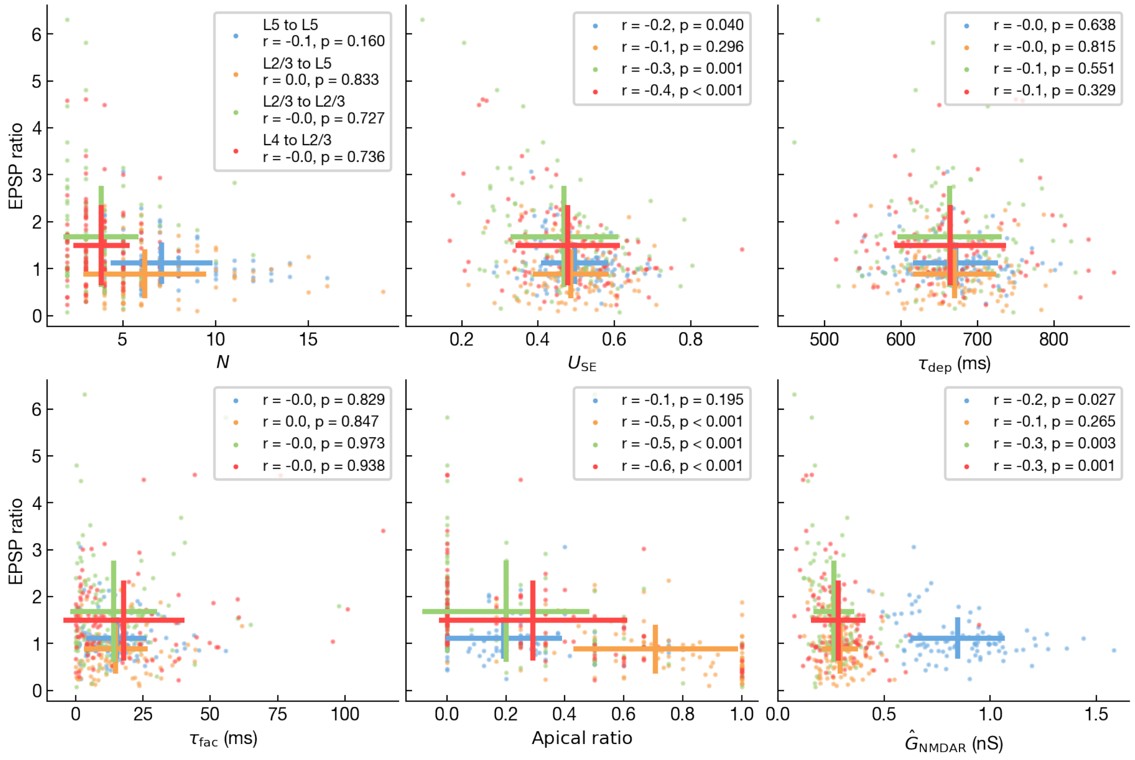

Figure A.8: Correlation between synaptic parameters and EPSP ratio for the 10 Hz, 10 ms STDP protocol across four connection types (L5-TTPC to L5-TTPC, blue, n = 100; L2/3-PC to L5-TTPC, orange, n = 99 due to an excluded outlier; L2/3-PC to L2/3-PC, green, n = 100; L4-PC to L4-PC, red, n = 100). Small filled circles represent plasticity outcomes of individual connections. Error bars indicate mean and standard deviation for each connection type. Spearman's correlation coefficient and corresponding p-value are reported in the legend. Same data as in Figure 6.

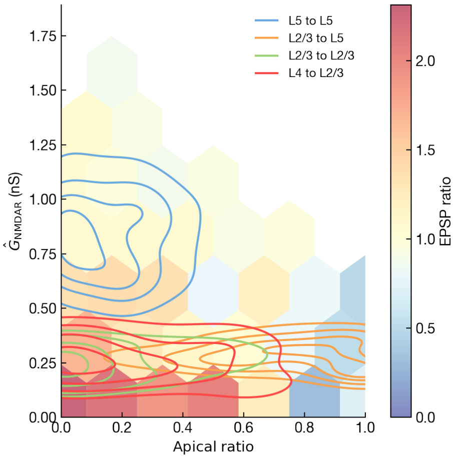

Figure A.9: Dependence of EPSP ratio on apical ratio and $\hat{g}_{\mathrm{NMDAR}}$ across four connection types (L5-TTPC to L5-TTPC, blue, n = 100; L2/3-PC to L5-TTPC, orange, n = 99 due to an excluded outlier; L2/3-PC to L2/3-PC, green, n = 100; L4-PC to L4-PC, red, n = 100). Hexagons are coloured according to the mean EPSP ratio of all connections of all types within their boundaries. Contours correspond to iso-proportions of the KDE of types specific connections at levels 0.9, 0.7, 0.5 and 0.3. Same data as in Figure 6.

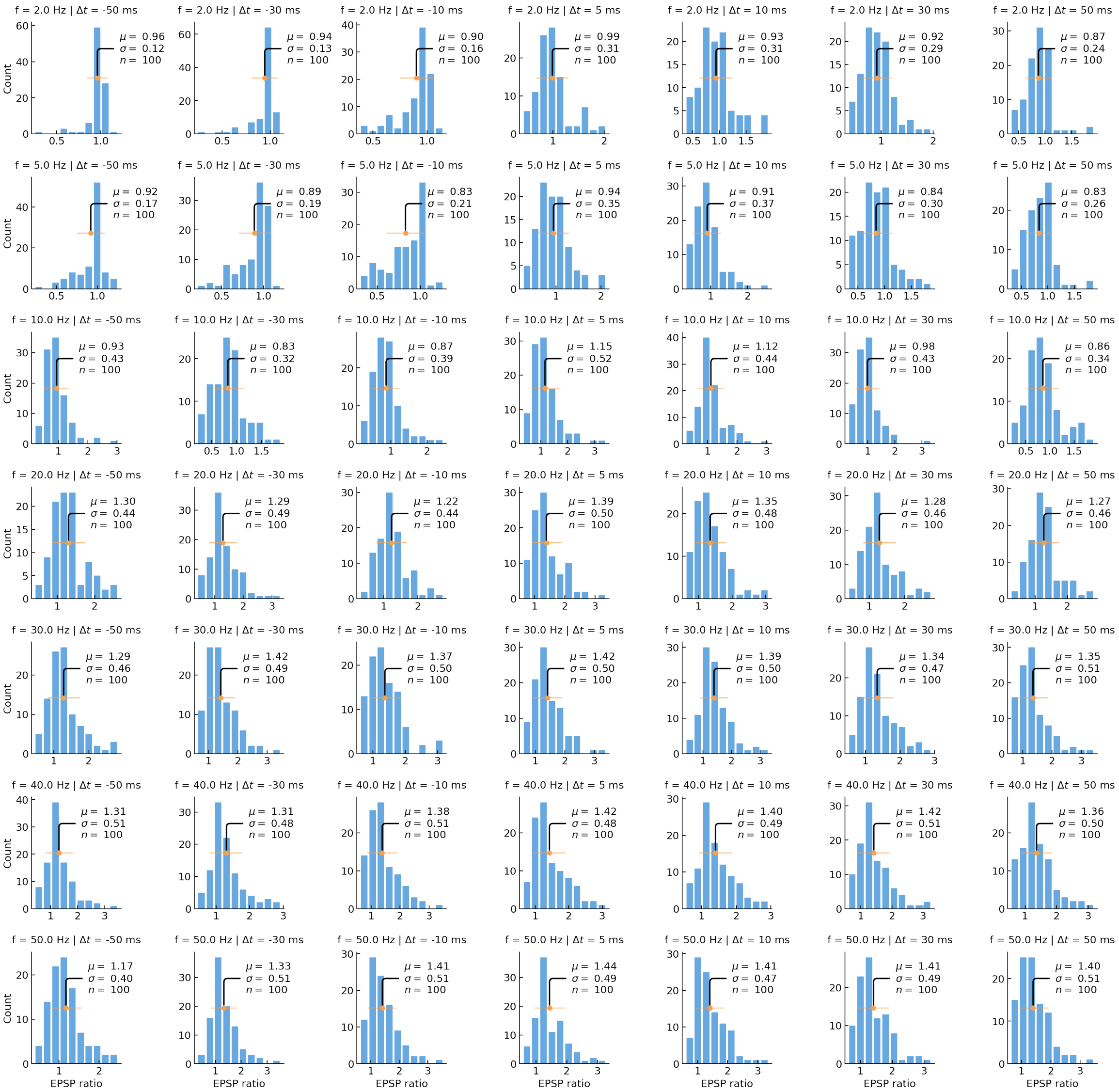

Figure A.10: Distribution of EPSP ratios for the *in silico* experiments between L5-TTPC to L5-TTPC connections shown in Figure 3e-f. Frequency and timing of the plasticity induction protocol is reported in the title of each panel. Orange error bars show mean (μ) and STD (σ) of the distribution. The total number of pairs (n) is reported in the annotations.

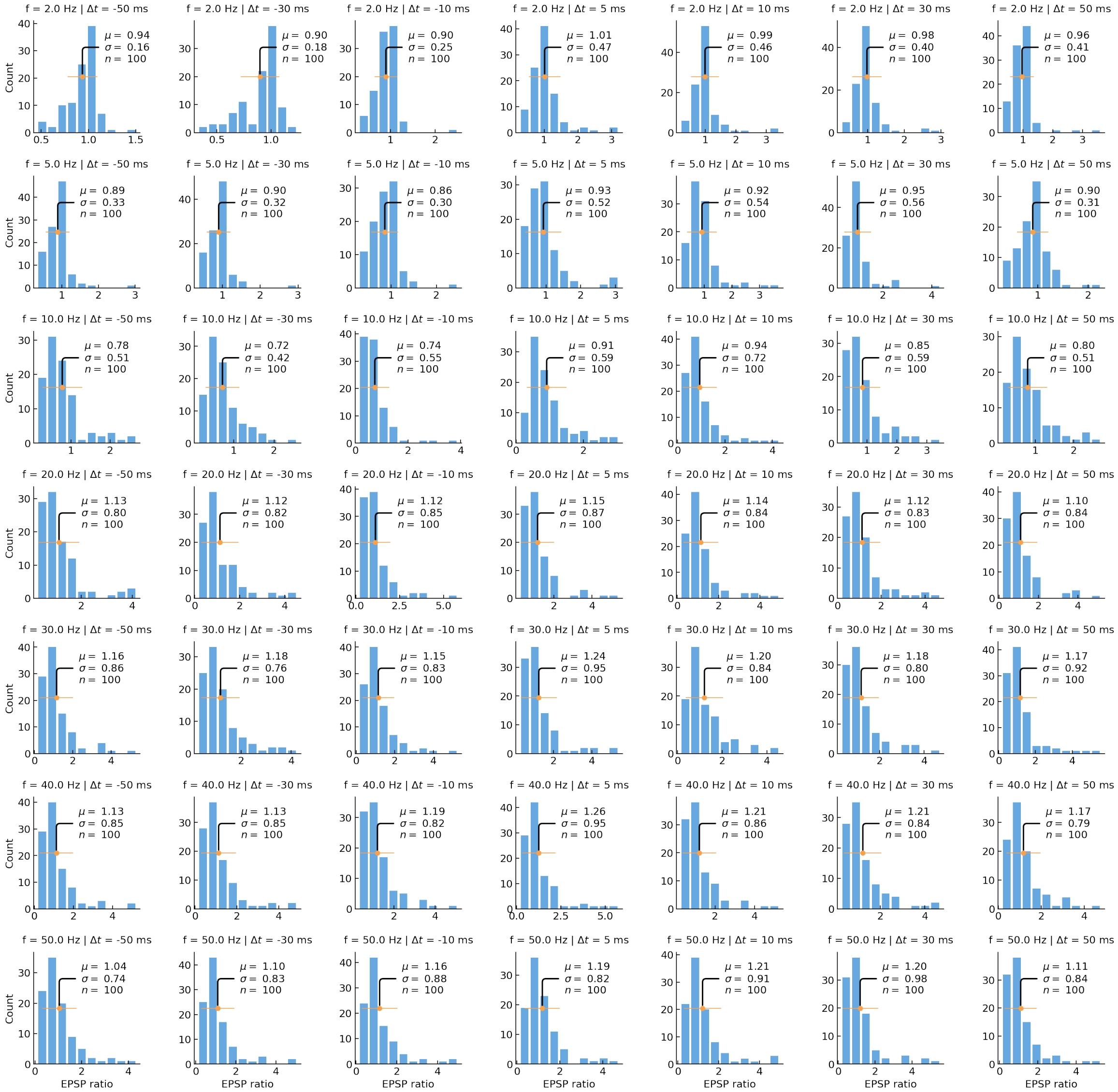

Figure A.11: Distribution of EPSP ratios for the *in silico* experiments between L2/3-PC to L5-TTPC connections shown in Figure 3i-l. Frequency and timing of the plasticity induction protocol is reported in the title of each panel. Orange error bars show mean (μ) and STD (σ) of the distribution. The total number of pairs (n) is reported in the annotations.

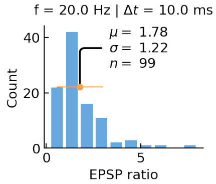

Figure A.12: Distribution of EPSP ratios for the *in silico* experiments between L2/3-PC to L2/3-PC connections shown in Figure 4c-d. Frequency and timing of the plasticity induction protocol is reported in the title of each panel. Orange error bars show mean (μ) and STD (σ) of the distribution. The total number of pairs (n) is reported in the annotations.

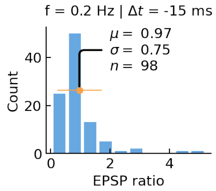

Figure A.13: Distribution of EPSP ratios for the *in silico* experiments between L2/3-PC to L2/3-PC connections shown in Figure 4f-g. Frequency and timing of the plasticity induction protocol is reported in the title of each panel. Orange error bars show mean (μ) and STD (σ) of the distribution. The total number of pairs (n) is reported in the annotations.

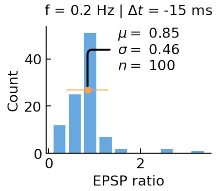

Figure A.14: Distribution of EPSP ratios for the *in silico* experiments between L4-PC to L2/3-PC connections shown in Figure 5. Frequency and timing of the plasticity induction protocol is reported in the title of each panel. Orange error bars show mean (μ) and STD (σ) of the distribution. The total number of pairs (n) is reported in the annotations.

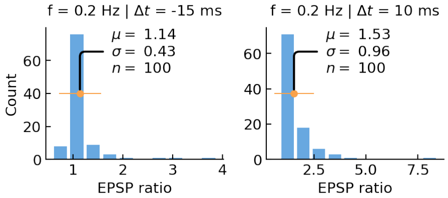

Figure A.15: Distribution of EPSP ratios for the *in silico* experiments between L4-PC to L2/3-PC connections shown in Figure 5, under the $\gamma_d = 0$ condition. Frequency and timing of the plasticity induction protocol is reported in the title of each panel. Orange error bars show mean (μ) and STD (σ) of the distribution. The total number of pairs (n) is reported in the annotations.

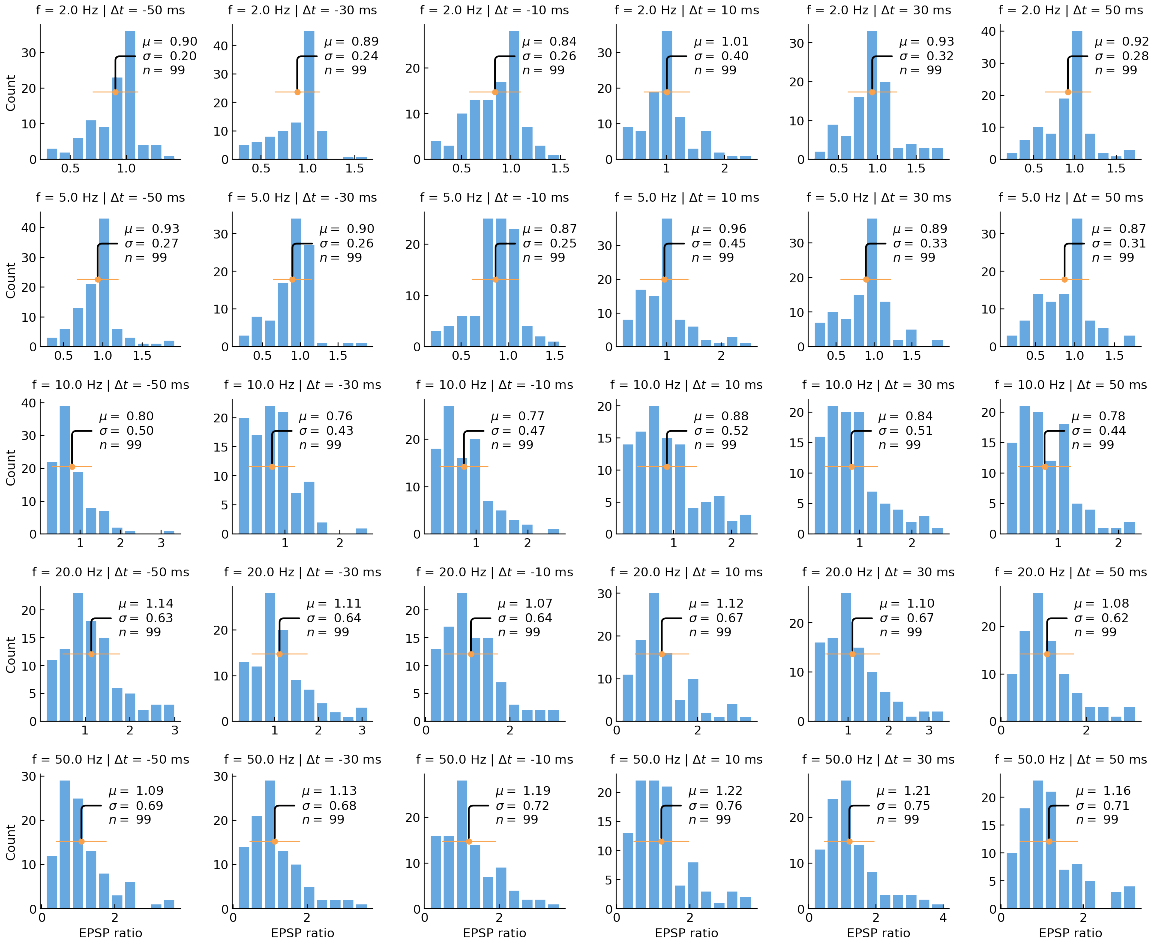

Figure A.16: Distribution of EPSP ratios for the *in silico* experiments between L2/3-PC to L5-TTPC connections shown in Figure 6. Frequency and timing of the plasticity induction protocol is reported in the title of each panel. Orange error bars show mean (μ) and STD (σ) of the distribution. The total number of pairs (n) is reported in the annotations.

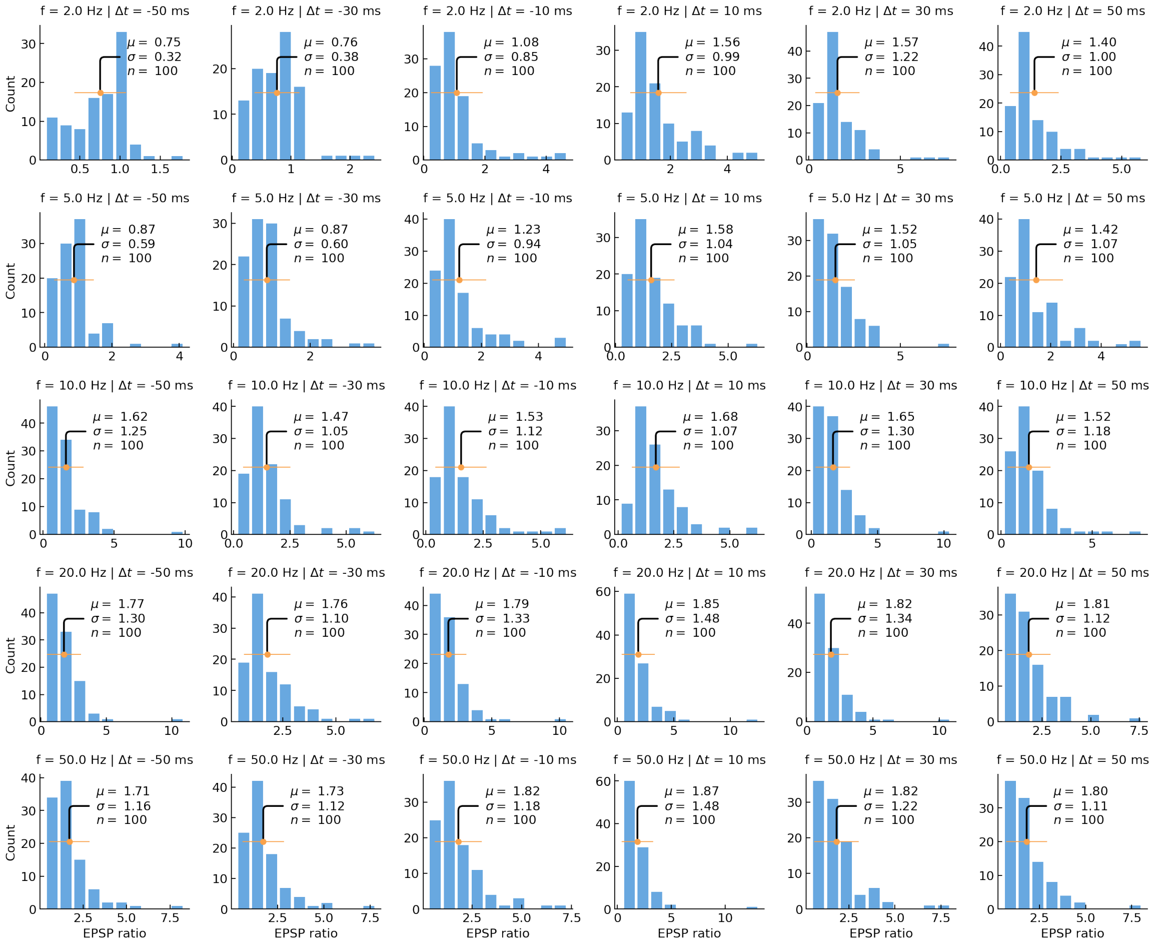

Figure A.17: Distribution of EPSP ratios for the *in silico* experiments between L2/3-PC to L2/3-PC connections shown in Figure 6. Frequency and timing of the plasticity induction protocol is reported in the title of each panel. Orange error bars show mean (μ) and STD (σ) of the distribution. The total number of pairs (n) is reported in the annotations.

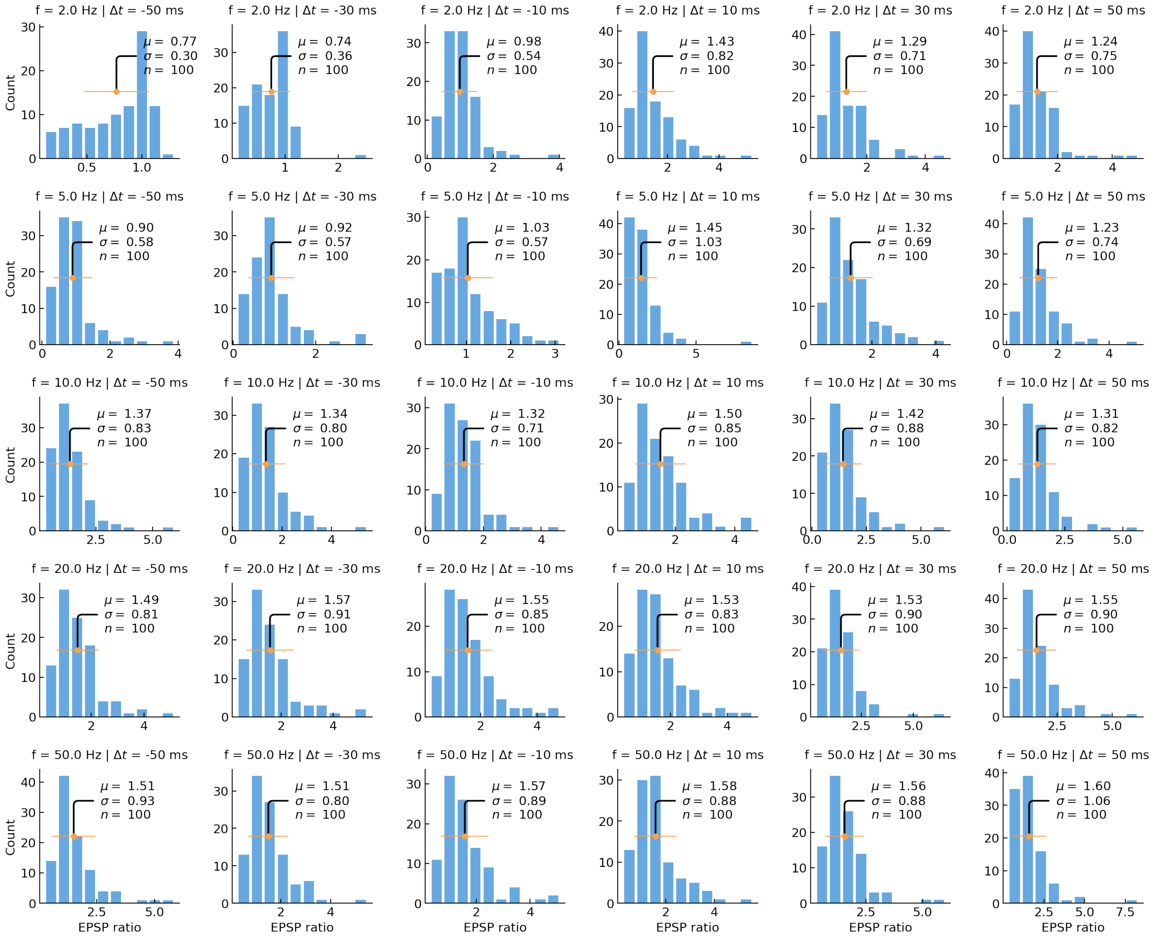

Figure A.18: Distribution of EPSP ratios for the *in silico* experiments between L4-PC to L2/3-PC connections shown in Figure 6. Frequency and timing of the plasticity induction protocol is reported in the title of each panel. Orange error bars show mean (μ) and STD (σ) of the distribution. The total number of pairs (n) is reported in the annotations.

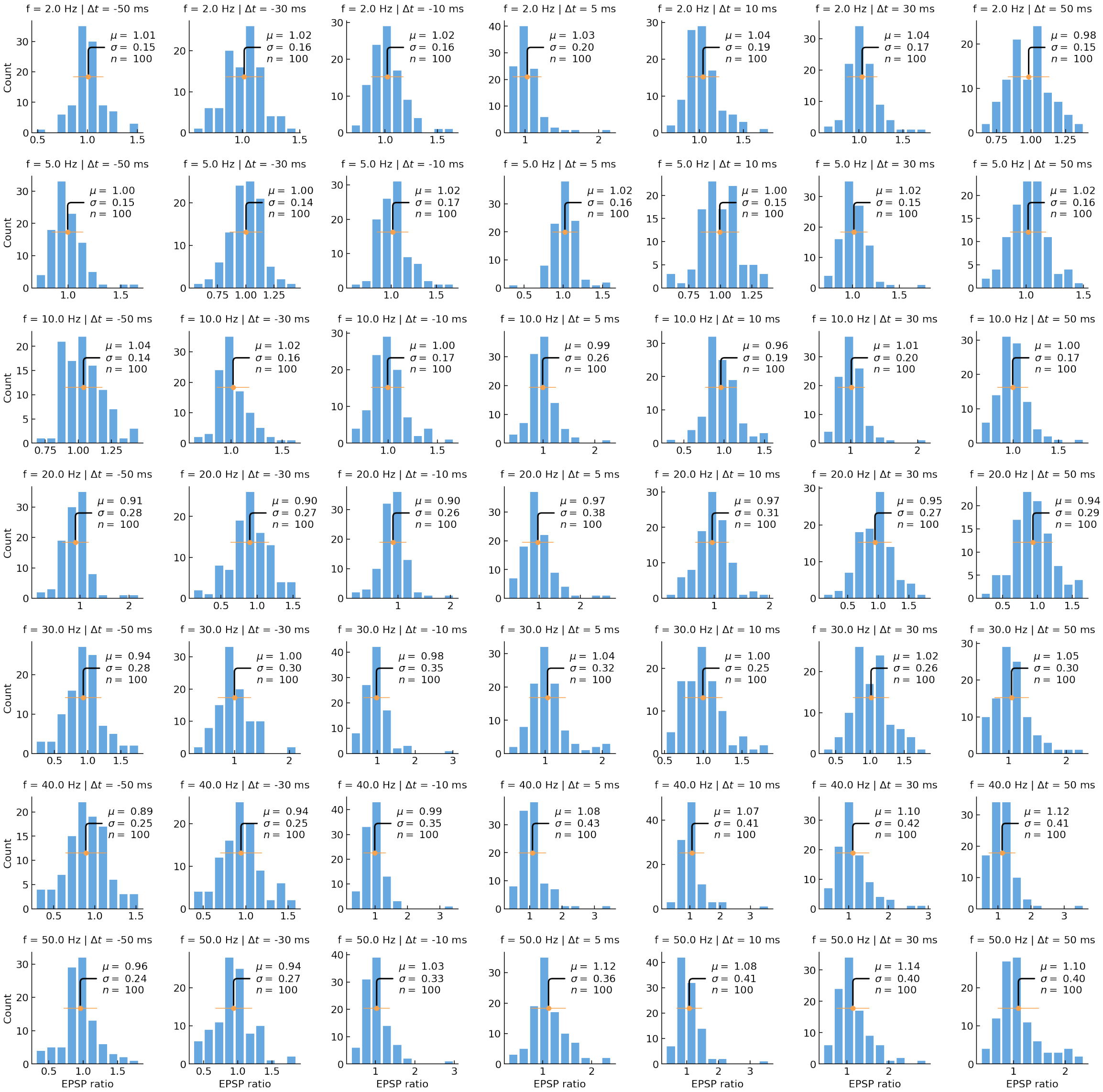

Figure A.19: Distribution of EPSP ratios for the *in silico* experiments between L5-TTPC to L5-TTPC connections shown in Figure 7, under the low calcium condition. Frequency and timing of the plasticity induction protocol is reported in the title of each panel. Orange error bars show mean (μ) and STD (σ) of the distribution. The total number of pairs (n) is reported in the annotations.

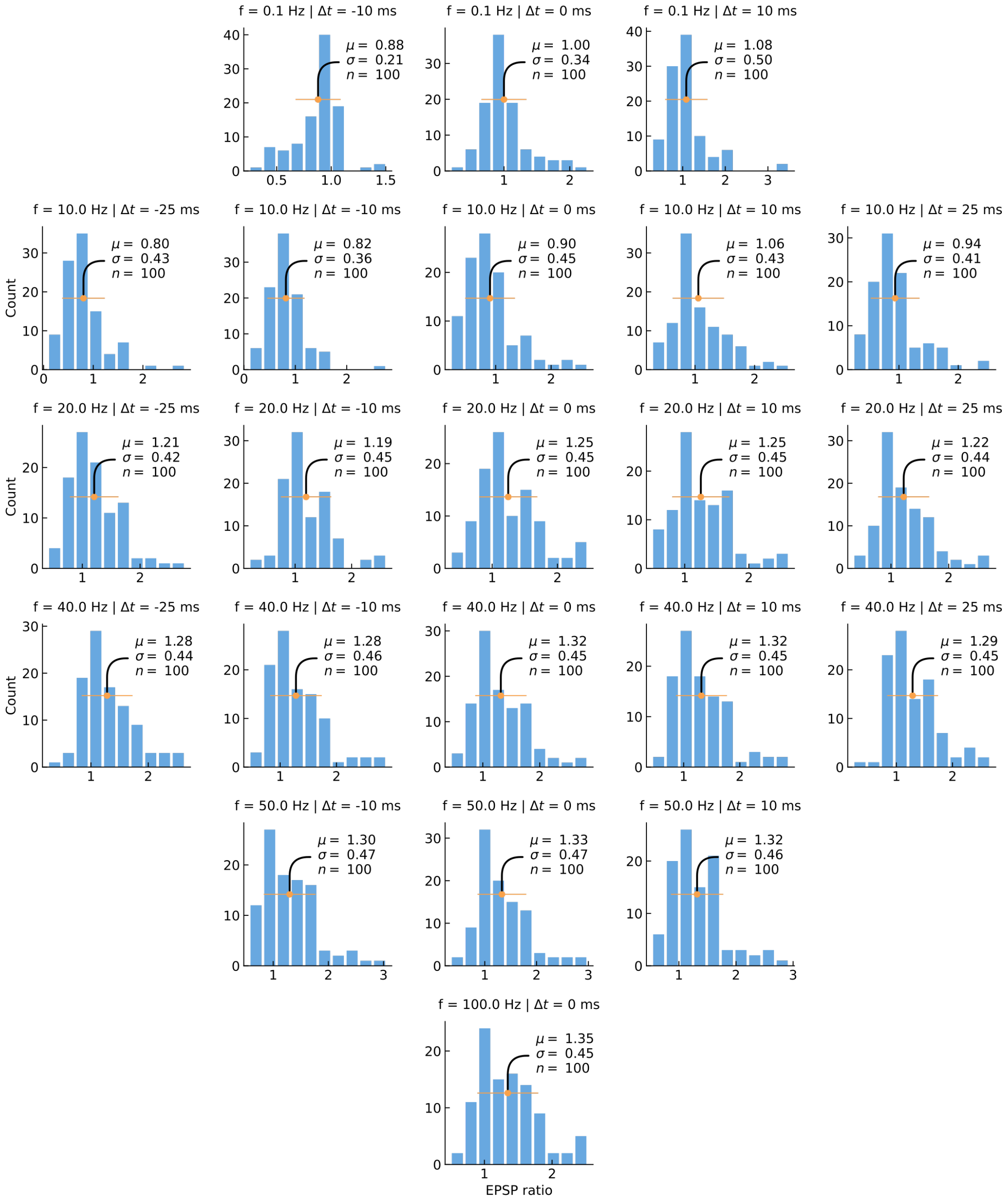

Figure A.20: Distribution of EPSP ratios for the *in silico* experiments between L5-TTPC to L5-TTPC connections shown in Supplementary Figure A.4. Frequency and timing of the plasticity induction protocol is reported in the title of each panel. Orange error bars show mean (μ) and STD (σ) of the distribution. The total number of pairs (n) is reported in the annotations.

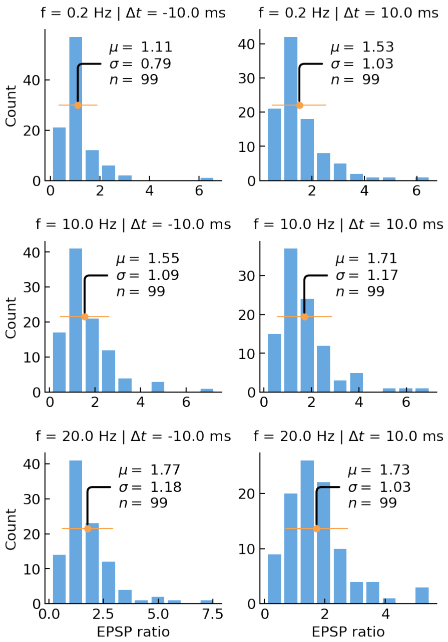

Figure A.21: Distribution of EPSP ratios for the *in silico* experiments between L2/3-PC to L2/3-PC connections shown in Supplementary Figure A.6. Frequency and timing of the plasticity induction protocol is reported in the title of each panel. Orange error bars show mean (μ) and STD (σ) of the distribution. The total number of pairs (n) is reported in the annotations.

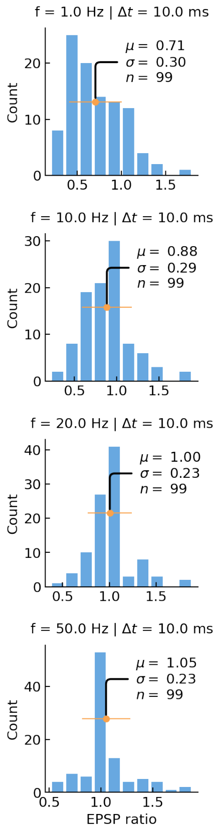

Figure A.22: Distribution of EPSP ratios for the *in silico* experiments between L4-SSC to L4-SSC connections shown in Supplementary Figure A.7. Frequency and timing of the plasticity induction protocol is reported in the title of each panel. Orange error bars show mean (μ) and STD (σ) of the distribution. The total number of pairs (n) is reported in the annotations.

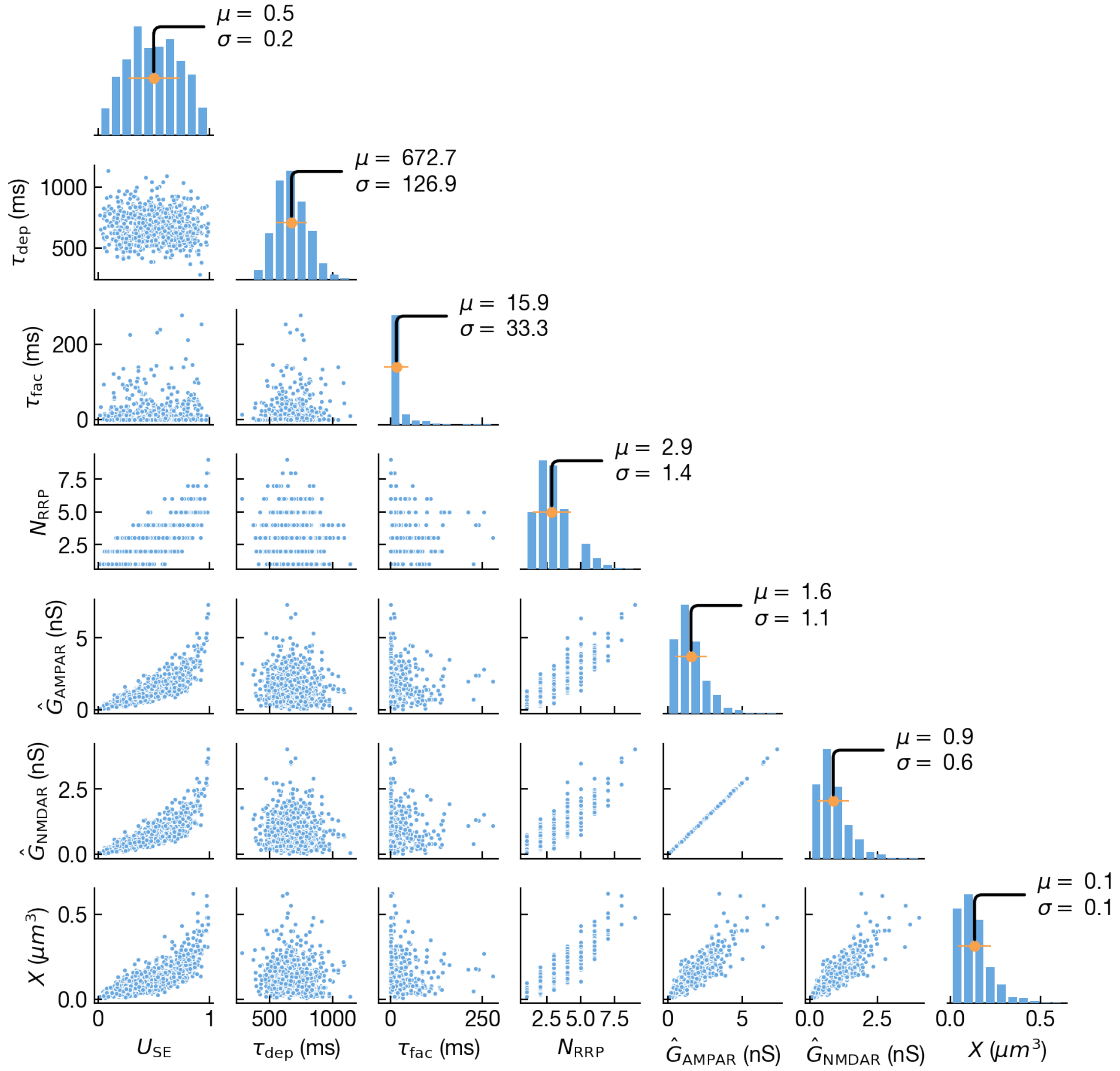

Figure A.23: L5-TTPC to L5-TTPC synaptic parameters distributions (main diagonal) and correlations (lower triangular matrix). Orange error bars show mean (μ) and STD (σ) of each parameter, $n = 710$.

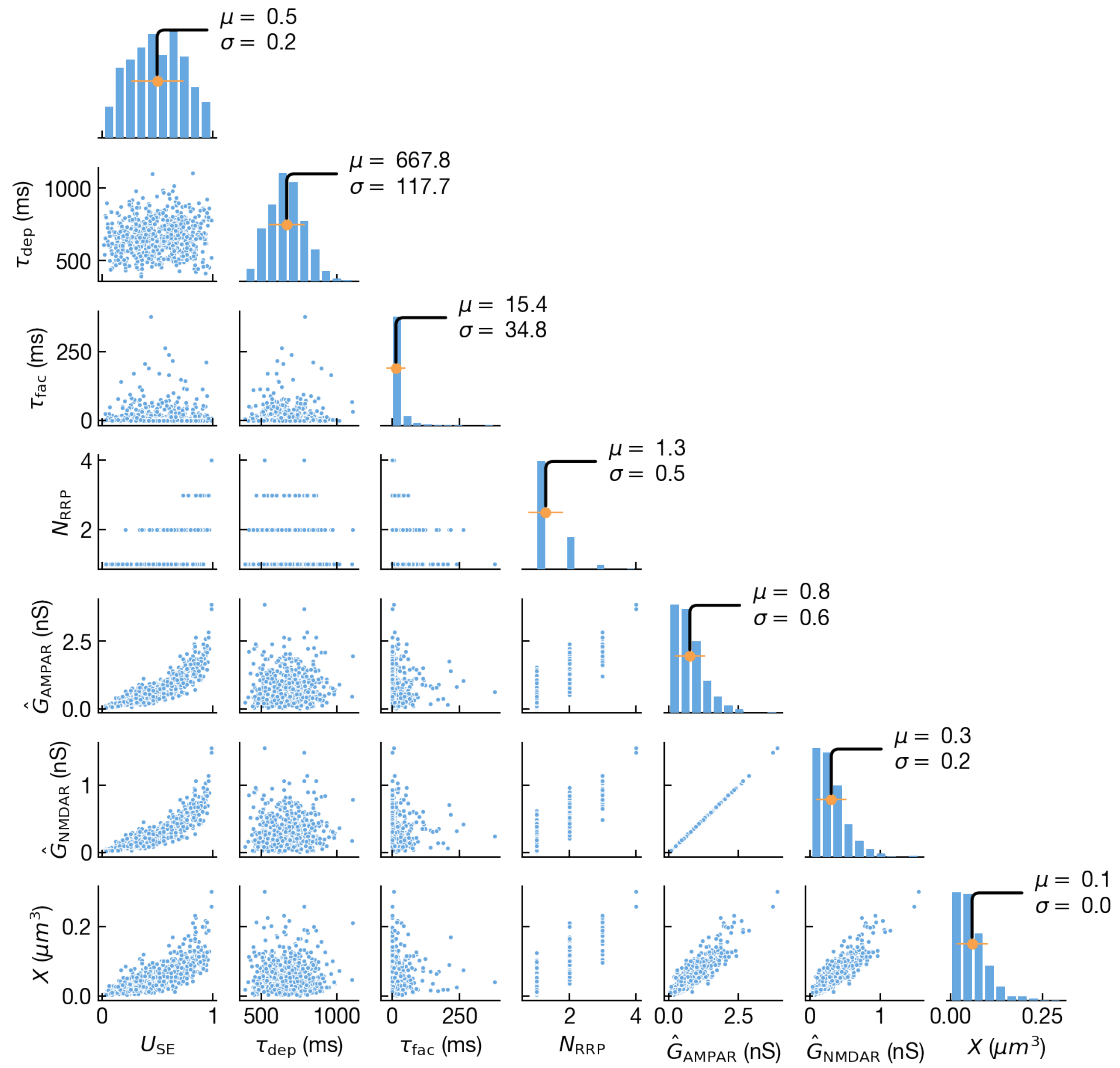

Figure A.24: L2/3-PC to L5-TTPC synaptic parameters distributions (main diagonal) and correlations (lower triangular matrix). Orange error bars show mean (μ) and STD (σ) of each parameter, $n = 649$.

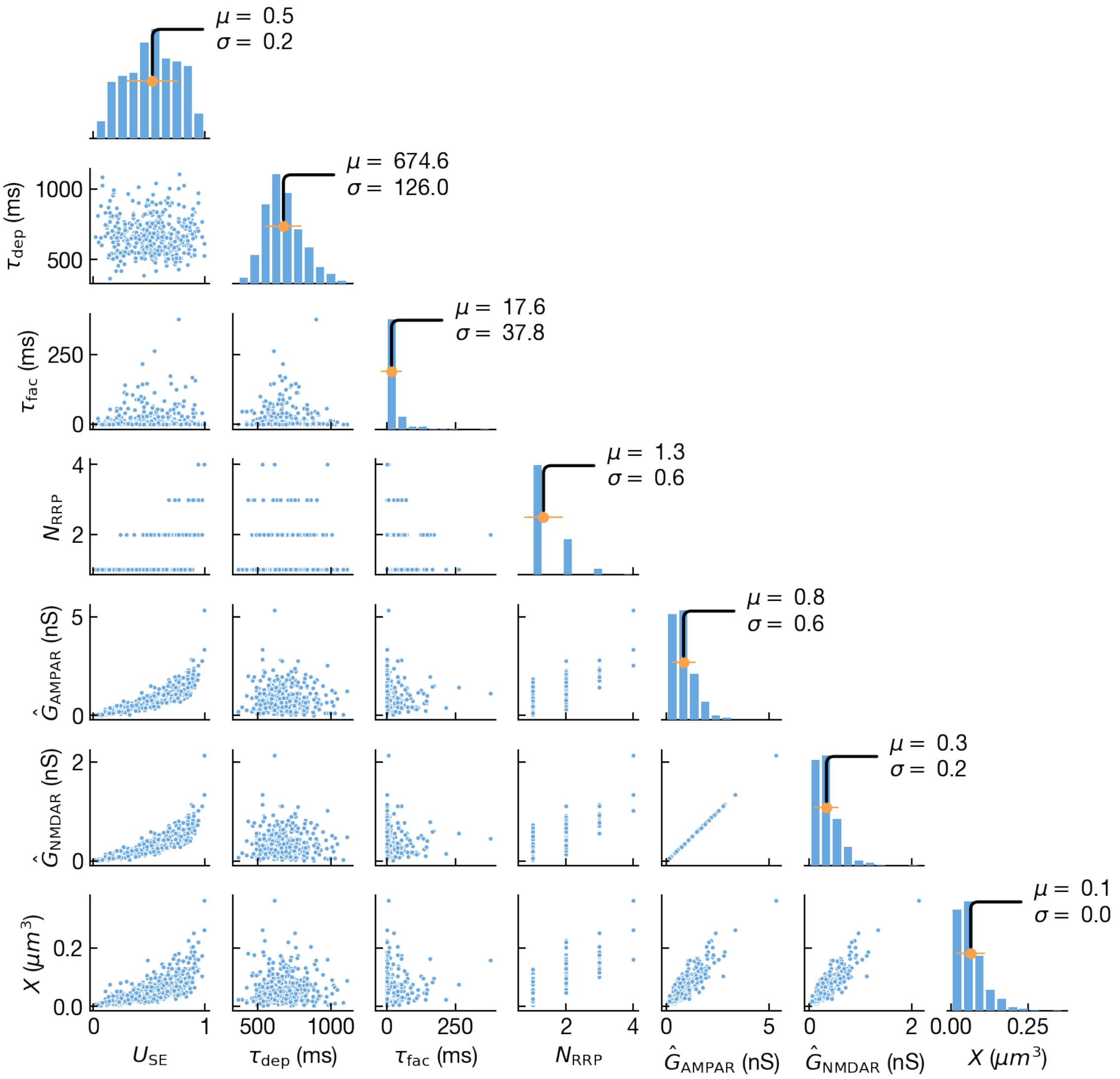

Figure A.25: L4-PC to L2/3-PC synaptic parameters distributions (main diagonal) and correlations (lower triangular matrix). Orange error bars show mean (μ) and STD (σ) of each parameter, $n = 422$.

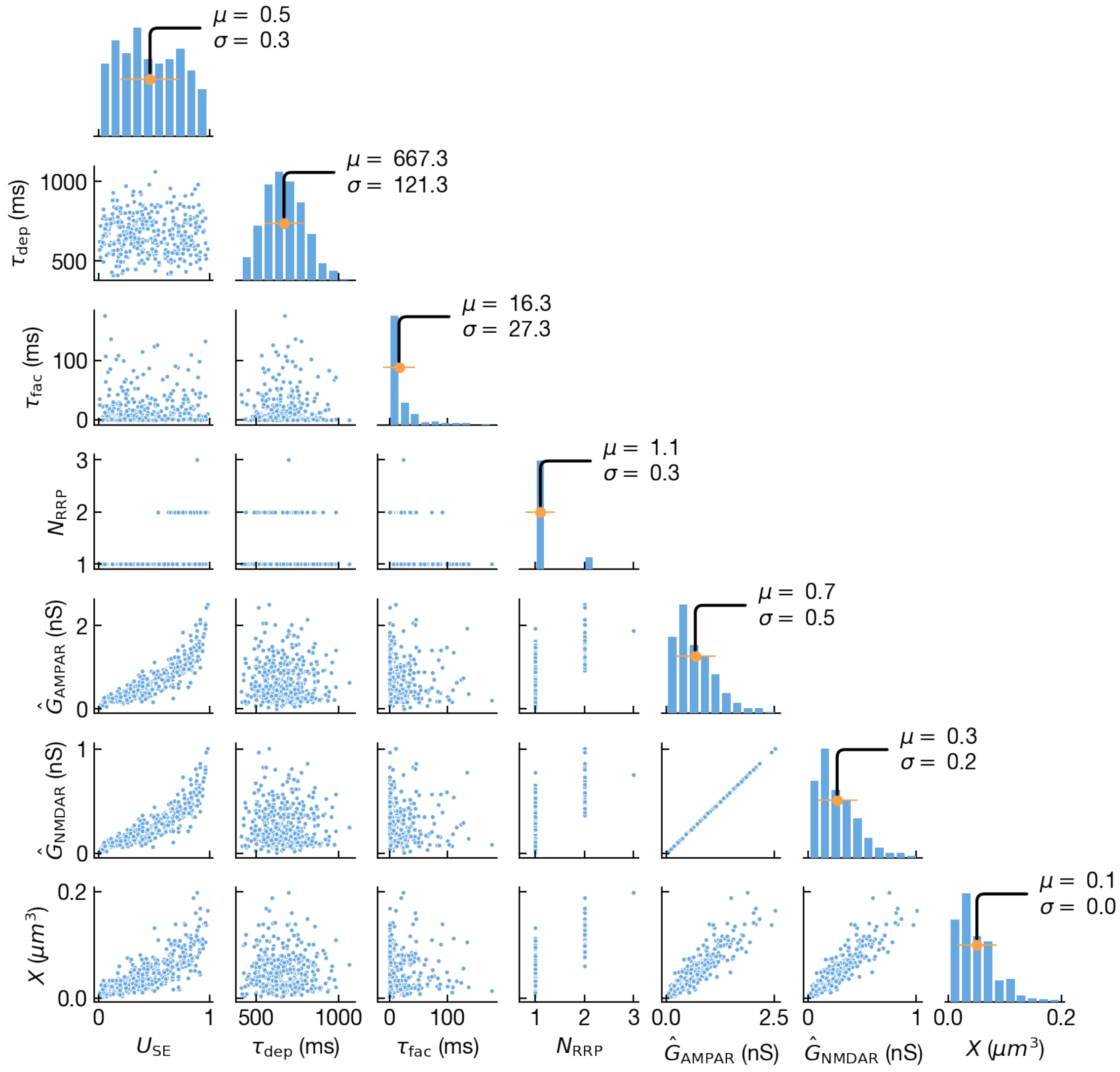

Figure A.26: L2/3-PC to L2/3-PC synaptic parameters distributions (main diagonal) and correlations (lower triangular matrix). Orange error bars show mean (μ) and STD (σ) of each parameter, $n = 369$.

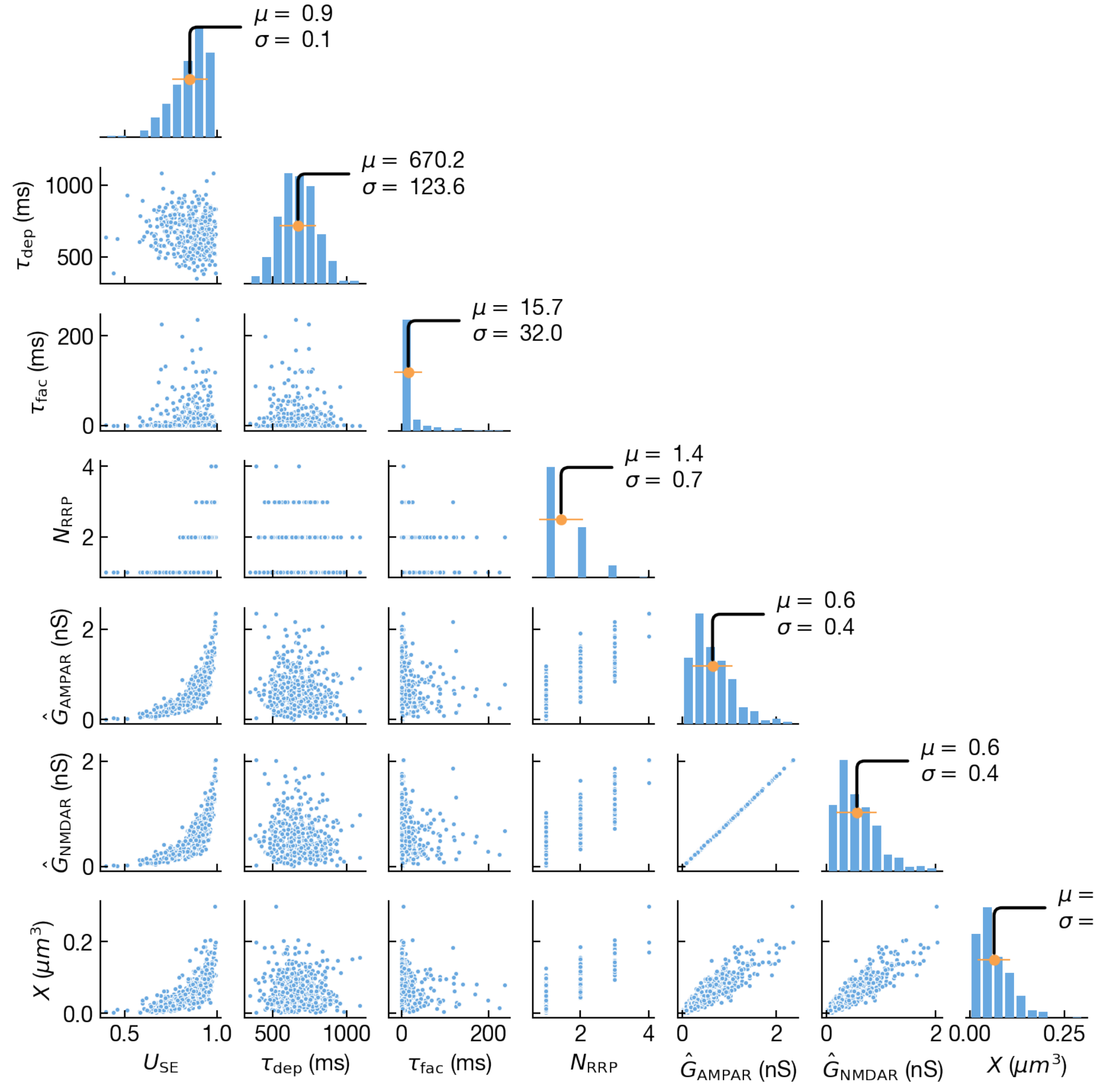

Figure A.27: L4-SSC to L4-SSC synaptic parameters distributions (main diagonal) and correlations (lower triangular matrix). Orange error bars show mean (μ) and STD (σ) of each parameter, $n = 458$.

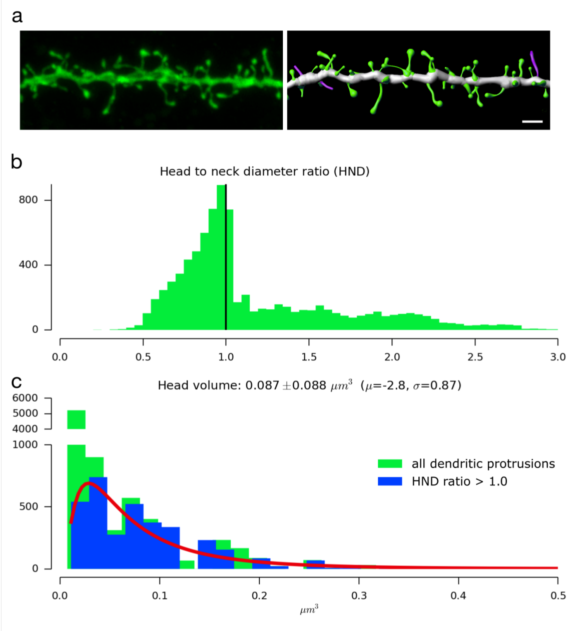

Figure A.28: Spine volume reconstruction. (**a**) Left: Confocal microscopy image showing a labeled basal dendrite from a layer 5 pyramidal neuron of the somatosensory cortex of the P14 rat. Right: Three-dimensional reconstruction of the dendritic shaft (white), spines (green) and filopodia (purple) shown on the left. Scale bar corresponds to 1.5 µm. (**b**) Head-to-neck diameter (HND) ratio. Dendritic protrusion showing a HND ratio of at least 1 (right of black line) were classified as spines and included in the analysis, while putative filopodia (left of black line) were excluded. (**c**) Head volume distribution of all dendritic protrusions (green), spines (blue) and the corresponding log-normal distribution fit (red). The data was obtained from 44 dendritic segments including 8423 dendritic spines which were individually reconstructed using the software Imaris (Bitplane AG, Zurich, Switzerland).

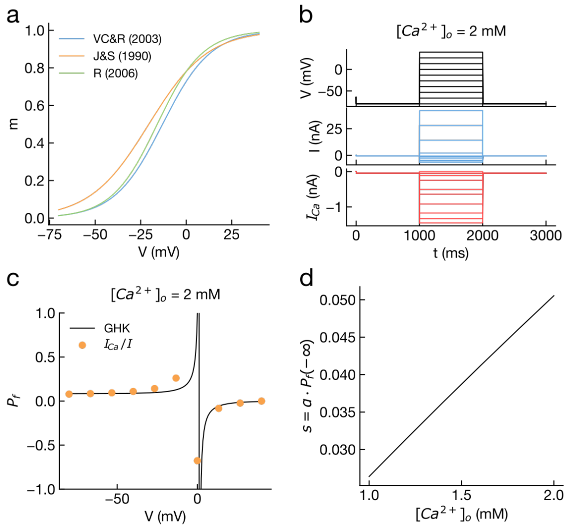

Figure A.29: Parameterization of NMDAR magnesium block and fractional calcium current. (**a**) Comparison of voltage dependence of the instantaneous magnesium block gating mechanism in Jahr & Stevens[8] (hippocampus), Vargas-Caballero & Robinson[9] (neocortex) and Rhodes[10] (theoretical). The parameterization of Vargas-Caballero & Robinson[9] was used in this work. (**b**) Simulation of NMDAR mediated currents for a range of holding voltages (top). Total current (middle) and calcium component of the current (bottom) were measured at steady state for $[Ca^{2+}]_o = 2$ mM. (**c**) Comparison of fractional calcium current, P_f, computed from simulated currents in (b; orange circles) with the expression of the same quantify derived in Schneggenburger *et al.*[11] from the GHK flux equation (black line). (**d**) Dependence of fractional calcium conductance, s, on extracellular calcium concentration for the range considered in this work.

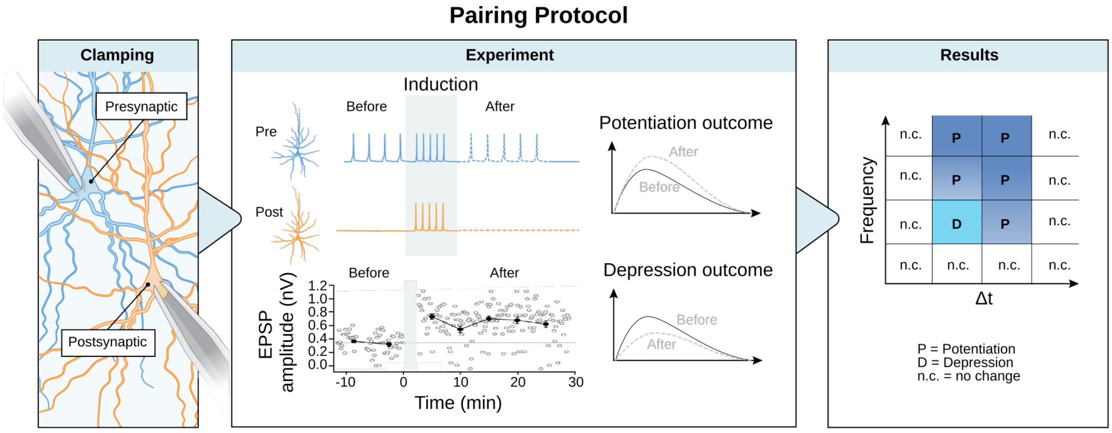

Figure A.30: A schematic description of a typical synaptic plasticity experiment in paired recording *in vitro*. Connected pairs of neurons (left) are stimulated by pairing the activity of the pre- and postsynaptic neuron. The stimulation protocol (center) includes three phases: an initial assessment of the connection strength, a plasticity-induction protocol where the activity of the two neurons is paired at a given frequency and time difference, a prolonged evaluation of connection strength. The ratio of mean EPSP amplitude after stimulation over initial mean EPSP amplitude is used as a measure of LTP/LTD as a function of stimulation frequency and/or spike-timing (right).

References

1. Sabatini, B. L., Oertner, T. G. & Svoboda, K. The Life Cycle of Ca2+ Ions in Dendritic Spines. *Neuron* **33,** 439–452. ISSN: 0896-6273. http://www.sciencedirect.com/science/article/pii/S0896627302005731 (2012) (Jan. 2002).

2. Markram, H., Lübke, J., Frotscher, M. & Sakmann, B. Regulation of Synaptic Efficacy by Coincidence of Postsynaptic APs and EPSPs. en. *Science* **275,** 213–215. ISSN: 0036-8075, 1095-9203. http://www.sciencemag.org/content/275/5297/213 (2014) (Jan. 1997).

3. Sjöström, P. J. & Häusser, M. A Cooperative Switch Determines the Sign of Synaptic Plasticity in Distal Dendrites of Neocortical Pyramidal Neurons. *Neuron* **51,** 227–238. ISSN: 0896-6273. http://www.sciencedirect.com/science/article/pii/S0896627306004715 (2015) (July 2006).

4. Sjöström, P. J., Turrigiano, G. G. & Nelson, S. B. Rate, Timing, and Cooperativity Jointly Determine Cortical Synaptic Plasticity. *Neuron* **32,** 1149–1164. ISSN: 0896-6273. http://www.sciencedirect.com/science/article/pii/S0896627301005426 (2012) (Dec. 2001).

5. Sjöström, P. J., Turrigiano, G. G. & Nelson, S. B. Multiple forms of long-term plasticity at unitary neocortical layer 5 synapses. *Neuropharmacology. LTP: Forty Unforgettable Years. A Festschrift in Honour of Professor Tim Bliss FRS* **52,** 176–184. ISSN: 0028-3908. http://www.sciencedirect.com/science/article/pii/S0028390806002310 (2016) (Jan. 2007).

6. Egger, V., Feldmeyer, D. & Sakmann, B. Coincidence detection and changes of synaptic efficacy in spiny stellate neurons in rat barrel cortex. En. *Nature Neuroscience* **2,** 1098. ISSN: 1546-1726. https://www.nature.com/articles/nn1299_1098 (2018) (Dec. 1999).

7. Zilberter, M. *et al.* Input Specificity and Dependence of Spike Timing–Dependent Plasticity on Preceding Postsynaptic Activity at Unitary Connections between Neocortical Layer 2/3 Pyramidal Cells. en. *Cerebral Cortex* **19,** 2308–2320. ISSN: 1047-3211, 1460-2199. http://cercor.oxfordjournals.org/content/19/10/2308 (2015) (Oct. 2009).

8. Jahr, C. E. & Stevens, C. F. Voltage dependence of NMDA-activated macroscopic conductances predicted by single-channel kinetics. en. *Journal of Neuroscience* **10,** 3178–3182. ISSN: 0270-6474, 1529-2401. http://www.jneurosci.org/content/10/9/3178 (2017) (Sept. 1990).

9. Vargas-Caballero, M. & Robinson, H. P. C. A Slow Fraction of Mg2+ Unblock of NMDA Receptors Limits Their Contribution to Spike Generation in Cortical Pyramidal Neurons. en. *Journal of Neurophysiology* **89,** 2778–2783. ISSN: 0022-3077, 1522-1598. http://jn.physiology.org/content/89/5/2778 (2017) (May 2003).

10. Rhodes, P. The Properties and Implications of NMDA Spikes in Neocortical Pyramidal Cells. en. *Journal of Neuroscience* **26,** 6704–6715. ISSN: 0270-6474, 1529-2401. http://jneurosci.org/content/26/25/6704 (2016) (June 2006).

11. Schneggenburger, R., Zhou, Z., Konnerth, A. & Neher, E. Fractional contribution of calcium to the cation current through glutamate receptor channels. *Neuron* **11,** 133–143. ISSN: 0896-6273. http://www.sciencedirect.com/science/article/pii/089662739390277X (2015) (July 1993).
