# Supplementary material for: A calcium-based plasticity model for predicting long-term potentiation and depression in the neocortex
Source: Nat Commun. 2022 Jun 1;13:3038. doi: 10.1038/s41467-022-30214-w (PMC9160074; doi:10.1038/s41467-022-30214-w)
Supplement: Supplementary file 3 — Reporting Summary [file 41467_2022_30214_MOESM3_ESM.pdf]

nature portfolio

Corresponding author(s): Giuseppe Chindemi

Last updated by author(s): Mar 16, 2022

Reporting Summary

Nature Portfolio wishes to improve the reproducibility of the work that we publish. This form provides structure for consistency and transparency in reporting. For further information on Nature Portfolio policies, see our Editorial Policies and the Editorial Policy Checklist.

Statistics

For all statistical analyses, confirm that the following items are present in the figure legend, table legend, main text, or Methods section.

n/a	Confirmed	
☐	☒	The exact sample size (*n*) for each experimental group/condition, given as a discrete number and unit of measurement
☐	☒	A statement on whether measurements were taken from distinct samples or whether the same sample was measured repeatedly
☐	☒	The statistical test(s) used AND whether they are one- or two-sided<br>*Only common tests should be described solely by name; describe more complex techniques in the Methods section.*
☐	☒	A description of all covariates tested
☐	☒	A description of any assumptions or corrections, such as tests of normality and adjustment for multiple comparisons
☐	☒	A full description of the statistical parameters including central tendency (e.g. means) or other basic estimates (e.g. regression coefficient) AND variation (e.g. standard deviation) or associated estimates of uncertainty (e.g. confidence intervals)
☐	☒	For null hypothesis testing, the test statistic (e.g. *F*, *t*, *r*) with confidence intervals, effect sizes, degrees of freedom and *P* value noted<br>*Give P values as exact values whenever suitable.*
☒	☐	For Bayesian analysis, information on the choice of priors and Markov chain Monte Carlo settings
☒	☐	For hierarchical and complex designs, identification of the appropriate level for tests and full reporting of outcomes
☐	☒	Estimates of effect sizes (e.g. Cohen's *d*, Pearson's *r*), indicating how they were calculated

Our web collection on statistics for biologists contains articles on many of the points above.

Software and code

Policy information about availability of computer code

Data collection	NEURON (v7.7) and the BBP simulation infrastructure.<br>Simulations can be reproduced using EModelRunner (v1.1.3; https://github.com/BlueBrain/EModelRunner) and the cell packages available on Zenodo at https://doi.org/10.5281/zenodo.6352774 (v2.1).<br>Model optimization performed using BluePyOpt (v1.8.88; https://github.com/BlueBrain/BluePyOpt).
Data analysis	Dendritic spines were individually reconstructed using Imaris (v7.6.5).<br>Analysis routines were written in Python (v3.8.5), and make use of standard scientific packages: Matplotlib (v3.4.3), SciPy (v1.5.2), NumPy (v1.19.1), Pandas (v1.3.5), Seaborn (v0.11.2), JupyterLab (v2.2.8).<br>Statistical analysis was performed using Python/SciPy (Welch's unequal variances t-test, two-sided) and R/ks (v3.6.3/v1.13.2, KDE test; see also R/fasano.franceschini.test v1.1.0 for an alternative implementation).

For manuscripts utilizing custom algorithms or software that are central to the research but not yet described in published literature, software must be made available to editors and reviewers. We strongly encourage code deposition in a community repository (e.g. GitHub). See the Nature Portfolio guidelines for submitting code & software for further information.

Data

Policy information about availability of data

All manuscripts must include a data availability statement. This statement should provide the following information, where applicable:

- Accession codes, unique identifiers, or web links for publicly available datasets
- A description of any restrictions on data availability
- For clinical datasets or third party data, please ensure that the statement adheres to our policy

Analyzed simulation data, the synapse model and optimization results are publicly available on Zenodo at https://doi.org/10.5281/zenodo.6352774 (v2.1).

Field-specific reporting

Please select the one below that is the best fit for your research. If you are not sure, read the appropriate sections before making your selection.

☒ Life sciences ☐ Behavioural & social sciences ☐ Ecological, evolutionary & environmental sciences

For a reference copy of the document with all sections, see nature.com/documents/nr-reporting-summary-flat.pdf

Life sciences study design

All studies must disclose on these points even when the disclosure is negative.

Sample size	Sample size was determined by progressively increasing the number of connected cell pairs in the training set, until the training results would be reproducible on an independently drawn set of connections of the same size.<br>We found that a sample size n = 100 provided satisfactory results at a reasonable computational cost.
Data exclusions	In silico connections with very small initial EPSP amplitude were on rare occasions excluded from the analysis, as such connections were not reported in the experimental datasets we are comparing against. All of such cases are discussed in the manuscript.
Replication	Part of the experiments were performed by G.C. and A.E. independently.<br>All experiments ran using the proprietary BBP software infrastructure were replicated using the open source EModelRunner.
Randomization	Pairs of connected PC cells were randomly sampled from a larger circuit model. Training, validation and testing were performed on different random samples.
Blinding	Blinding was not relevant for this study as neuron pairs sampling was randomized, as described in the manuscript.

Reporting for specific materials, systems and methods

We require information from authors about some types of materials, experimental systems and methods used in many studies. Here, indicate whether each material, system or method listed is relevant to your study. If you are not sure if a list item applies to your research, read the appropriate section before selecting a response.

Materials & experimental systems

n/a	Involved in the study
☒	☐ Antibodies
☒	☐ Eukaryotic cell lines
☒	☐ Palaeontology and archaeology
☒	☐ Animals and other organisms
☒	☐ Human research participants
☒	☐ Clinical data
☒	☐ Dual use research of concern

Methods

n/a	Involved in the study
☒	☐ ChIP-seq
☒	☐ Flow cytometry
☒	☐ MRI-based neuroimaging
